# *Mycoplasma* Biofilms: Characteristics and Control Strategies

**DOI:** 10.3390/microorganisms13081850

**Published:** 2025-08-07

**Authors:** Jingyi Liang, Baoyi Deng, Weihuo Li, Jingjing Qi, Yangshuo Li, Xueyan Wang, Ming Li, Hong Yang, Nan Zhang

**Affiliations:** 1 School of Animal Science and Technology, Foshan University, Foshan 528225, China; jingyi_leung@outlook.com (J.L.); dby1143687918@outlook.com (B.D.); lwh2287495671@outlook.com (W.L.); 18738430717@163.com (Y.L.); xueyanwang157@gmail.com (X.W.); 15772198794@163.com (M.L.); yhong007@fosu.edu.cn (H.Y.); 2 Shanghai Veterinary Research Institute, The Chinese Academy of Agricultural Sciences (CAAS), 518 Ziyue Road, Shanghai 200241, China; qijingjing@shvri.ac.cn

**Keywords:** *Mycoplasma*, biofilm, drug resistance, virulence

## Abstract

The *Mycoplasmataceae* are a family of bacteria that typically cause respiratory, arthritic, and genitourinary disease in humans. *Mycoplasma* spp. of animal origin are also the causative agents of porcine wheezing disease, chronic respiratory disease and arthritis in chickens and other conditions. These diseases have a significant impact on public health and the economic development of livestock breeding. Clinical prevention and treatment of *mycoplasma* infections is primarily dependent on the use of antibiotics. However, inappropriate and excessive use of antimicrobials has enabled resistance development that has become a significant clinical concern. *Mycoplasma* are also robust biofilm producers, and this process is a major factor for the persistence of these infections, especially in conjunction with common antibiotic resistance mechanisms, including target gene mutations and the action of efflux pumps. A *mycoplasma* biofilm refers to a structured and stable microbial community formed by *Mycoplasma* spp. adhering to biological or non-biological surfaces under suitable conditions and secreting extracellular polymers (EPS) such as polysaccharides. This process allows the microorganisms to adapt to their surrounding environment and survive during the growth process. These biofilms render bacteria more resistant to antimicrobials than planktonic bacteria, resulting in biofilm-associated infections that are more challenging to eradicate and more likely to recur. The current study reviews progress from the fields of biofilm formation, structure and identification, correlations between biofilms and drug resistance and virulence as well as methods of biofilm prevention and control. Our aim was to provide a reference basis for the subsequent in-depth understanding of the research of *mycoplasma* biofilms.

## 1. Biological Characteristics of *Mycoplasma*

**Figure 1:**
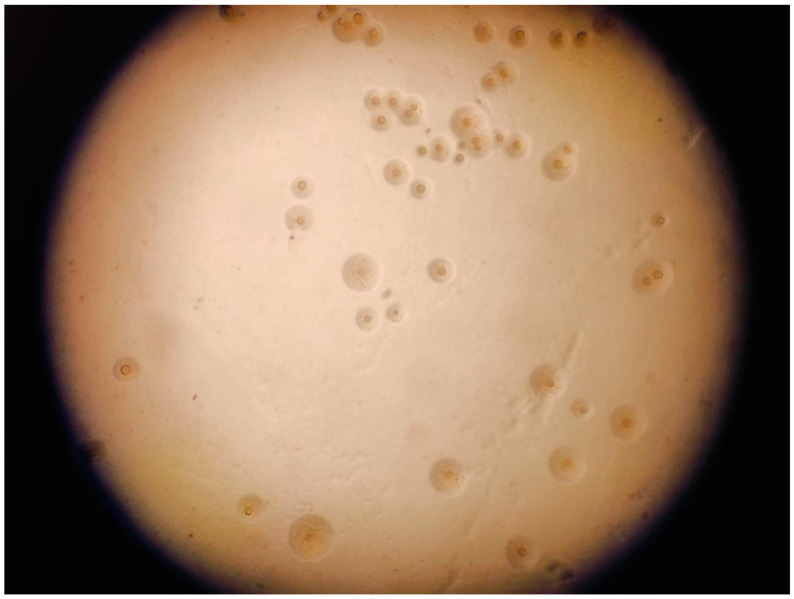
Fried egg colonies of *Mycoplasma gallisepticum*.

*Mycoplasma* spp. are prokaryotes (class Mollicutes) that are characterized by the lack of a cell wall, a small genome (500–2000 kb), and a pleomorphic morphology (spherical, filamentous, or irregular) with diameters of 100–300 nm. Their reduced size and structural flexibility enable them to pass through 0.22 µm sterilizing-grade membrane filters, a feature that complicates containment in laboratory and clinical environments [1]. *Mycoplasma* have nutritional requirements that differ from most other bacteria due to their lifestyle that is tightly linked to the host. For instance, cultures require supplementation with 10–20% human or animal serum to provide essential cholesterol needed for their cell membranes. Additionally, the optimum pH for growth is 7.8–8.0 and they cannot survive at pH < 7.0. Most are facultative anaerobes and some strains grow better when 5% CO_2_ is added at the time of initial isolation. They form smooth, round, transparent colonies of uniform size and roundness on solid plates (fried egg morphology) (Figure 1) [2]. The absence of a cell wall precludes any effectiveness of β-lactam and glycopeptide antibiotics or fosfomycin [3].

*Mycoplasma* are widely found in nature in humans, animals, and plants, and there are a wide variety of extant species. The first *mycoplasma* was isolated by Nocard in 1898 and was classified as *Mycoplasma mycoides* in 1967 [4]. Subsequently, *Mycoplasma* spp. have been isolated from the mucosal surfaces of the respiratory and genital tracts in both humans and animals. These bacteria are highly prevalent, and this has serious implications for both human and animal health. They are important animal pathogens and can cause serious disease in cattle, pigs, and poultry and cause chronic infections in humans and animals, including pneumonia [5,6,7,8], chronic bronchitis [9], arthritis [10,11,12], mucositis [13,14], chorioamnionitis [15], and reproductive disorders [16,17,18,19]. *Mycoplasma pneumoniae* is a significant causative agent of respiratory disease in children, resulting in upper and lower respiratory tract infections. The clinical presentation is characterized by coughing, headache, wheezing, and other non-specific manifestations that are commonly observed in children [6,20]. The presence of *Mycoplasma genitalium* is associated with an increased risk of urethritis and proctitis in men and cervicitis and pelvic inflammatory disease in women. These conditions are linked to an elevated risk of preterm labor, miscarriage, and infertility [21]. *Mycoplasma hyopneumoniae* is the primary pathogen of *Mycoplasma pneumonia* of swine as well as porcine respiratory disease syndrome. *M. hyopneumoniae* can cause chronic respiratory symptoms such as coughing and wheezing in pigs and make the animals highly susceptible to secondary infection with other viral and bacterial respiratory pathogens [22,23]. *Mycoplasma synoviae* is the causative agent of respiratory tract disease in poultry that is manifested by airsacculitis and joint disease. The latter is characterized by inflammatory exudative bursitis or tarsal arthritis. Additionally, *M. synoviae* has been linked to decreased egg production and abnormal eggshell formation [24].

## 2. Biofilms

A biofilm is defined as a structured community of microorganisms attached to a surface and embedded in its own secreted extracellular polymeric substance (EPS) matrix [25]. Its extracellular polymers form the material basis of the biofilm that includes as its primary components EPS, proteins, and extracellular DNA (eDNA) [26] that provide structural integrity to the biofilm (Table 1). Microbial communities typically account for 10–25% of the biofilm volume, and the remaining (75–90%) consists of EPS [27]. Adhesion–cohesion, scaffolding, mechanical stability, and protection are the most prominent features of the EPS component [28] and play a crucial role in protection from antibiotics and host immunity [29,30]. The EPS barrier effect makes bacteria less susceptible to antimicrobial agents and results in biofilm-forming *Mycoplasma* strains that have elevated levels of heat and desiccation resistance [31,32].

Planktonic *mycoplasma* adhere to biotic or abiotic surfaces such as mucosal membranes and implanted biomaterials. These biofilms represent a survival strategy under nutrient-limited conditions [38,39,40]. Numerous *Mycoplasma* species, including *M. pneumoniae* [41,42], are capable of forming biofilms in vivo and in vitro (Table 2).

### 2.1. Formation and Structure of Biofilms

The formation of *Mycoplasma* spp. biofilms is a dynamic and complex process, and like other bacteria, the initial stages are characterized by adhesion to host tissues or inanimate surfaces (such as glass). This is accomplished via its variable surface antigen (Vsa) proteins present on the surface [41]. The length and structure of Vsa proteins affect their adhesion ability. Short Vsa proteins facilitate biofilm formation, while long Vsa proteins may inhibit adhesion by steric hindrance and allow a planktonic lifestyle [52]. After initial adhesion, *mycoplasma* cells begin to secrete extracellular polymers (polysaccharides, lipids, DNA, or proteins) to form an EPS extracellular matrix, which surrounds the cells together and allows surface adhesion [34]. This aggregation forms microcolonies that are the basis of the biofilm structure. Over time, microcolonies develop into mature biofilms, characterized by complex three-dimensional structures that include honeycomb- and tower-like structures. These features are connected via the extracellular matrix to form channels that facilitate the transport of nutrients and the removal of waste [27]. As the biofilm ages, the nutrients within the biofilm are insufficient to support the internal community, and a large amount of waste and toxins accumulate. Some *Mycoplasma* spp. also express nucleases and glycosidases to degrade EPS, resulting in transformation into a free state for secondary infection [31]. Quorum sensing (QS) plays an important role in the process of biofilm development. QS is a microbial communication mechanism that coordinates the group behavior between microbial cells by producing and detecting signal molecules called autoinducers (AIs), influencing virulence gene expression, toxin production, and biofilm formation [53,54]. Although the genome of *mycoplasma* is relatively small and lacks a complex gene-regulation system, some *mycoplasmas* may still have a quorum sensing mechanism. For example, studies have shown that the biofilm formation of *Mycoplasma* genitalium is associated with the quorum sensing metabolic pathway, in which the protein translocase subunit is involved, and the biofilm formation contributes to the antibiotic tolerance of this *mycoplasma* [55].

The mechanisms of biofilm formation vary depending on species and environmental conditions. For example, particular strains of *M. bovis* form robust biofilms in vitro, while others do not, and this has been linked to selective adhesin expression [52]. In the case of *M. bovis*, biofilm formation ability is correlated with molecular type identified via amplified fragment length polymorphism (AFLP) and random amplified polymorphic DNA (RAPD). In particular, subgroup B are strong biofilm formers, while subgroup A are poor biofilm formers [31]. Additionally, *M. bovis* biofilm formation is significantly enhanced when co-cultured with the Gram-positive *Trueperella pyogenes*, and the morphological characteristics of these biofilms resemble those formed during in vivo infections [56]. The polymicrobial interactions between these two pathogens induce biofilm formation that increases resistance to antimicrobial agents and thereby exacerbates the progression of chronic pneumonia.

**Figure 2:**
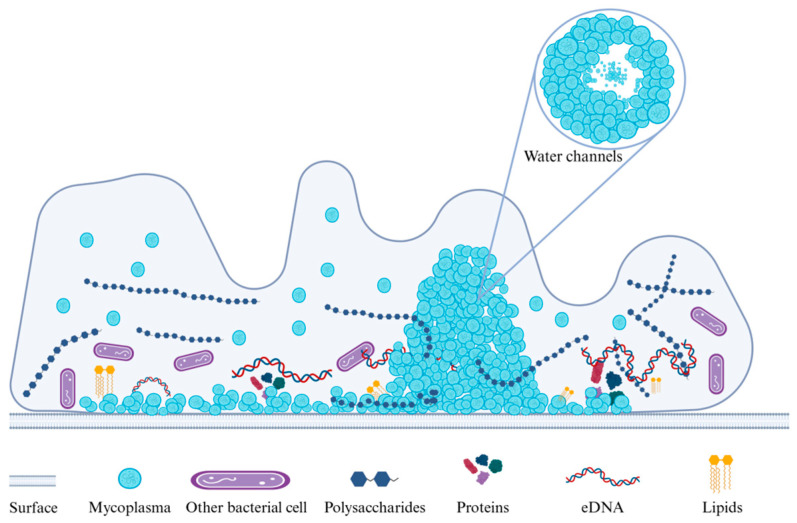
The volcano-like biofilm structure of *M. pneumoniae* is composed of densely arranged cells closely connected via EPS. Sparse water channels are present in the biofilm interior that promote the exchange of nutrients and waste and support the growth and maintenance of the biofilm.

Multiple experiments have also demonstrated that biofilm structures are diverse, and this phenotypic heterogeneity is most likely the result of differences in gene expression [57]. For example, *M. pneumoniae* can form volcano-like biofilm structures [41] (Figure 2), while *M. synoviae* biofilms exhibit mushroom- and tower-like structures [46]. *M. pulmonis* biofilms formed on tracheal epithelia in vivo are also tower-like structures [49].

Surface materials can significantly affect bacterial adhesion such that biofilm morphologies are dependent on surface material characteristics [58,59]. For example, *M. gallisepticum* biofilms exhibit lichen-like and ice-arabesque structures on glass coverslips and present cupola- or igloo-shaped structures on plastics [50]. EPS also can determine structures, and *M. genitalium* biofilms contain high levels of N-acetylglucosamine, indicating that EPS formation can be species-specific and may affect the final biofilm structure [29]. Polysaccharides are key components of the ECM and play the primary supporting role in the membrane structure. *Mycoplasma*–polysaccharide interactions are also influenced by type and genetic strain differences that also affect structure [42]. In addition, the presence of eDNA can promote the stability of biofilm, and biofilms formed in vitro involve large cell variants that contribute to eDNA release and enable *M. hyopneumoniae* to form biofilms on non-biological surfaces [26,47]. Biofilm formation and structures are influenced by a variety of factors, and understanding these processes is essential for interpreting the complexity and diversity of *mycoplasma* biofilms and developing clinical treatment methods.

### 2.2. Methods for the Identification of the Biofilm

#### 2.2.1. Staining Methods

Crystal Violet is a commonly used biofilm staining agent that can bind to and stain cells in the biofilm, thereby quantifying the relative amount of biomembrane formation through light absorption (OD value) or observing the structural characteristics of the biomembrane through microscopic imaging [60]. Although Crystal Violet can stain both living and dead bacteria, these can be distinguished by the MTT metabolic assay.

The Congo red staining method has the characteristics of rapid reaction, simple operation, and stable staining, which is another method for detecting biofilms in vitro. Its principle is that Congo red can combine with extracellular polymers to form dry red–brown crystals. This method can identify the presence or absence of biofilms, as well as quantify the polysaccharides and cellulose in biofilms [61]. However, Kang et al. [46] found that when staining the biofilms formed by *M. synoviae*, the sensitivity of Congo red staining is not as high as that of crystal violet staining.

#### 2.2.2. Imaging Assays

Biofilm can be microscopically imaged, and this provides a qualitative and quantitative detection method. Most commonly, scanning electron microscopy (SEM), focused ion beam (FIB), and confocal laser scanning microscopy (CLSM) are used for the direct observation of the structure and morphology of biofilms. The SEM provides a detailed view of the biofilm and its extracellular polymers and enables detection of towers and channels. The principal constraints associated with SEM are sample dehydration to ensure their suitability for vacuum operations, and this cumbersome process may potentially compromise the structural integrity of the matrix [62]. The FIB microscope is an effective tool for examining the internal structure of biofilms and eliminates the need for time-consuming pretreatment procedures. Its principle is to remove the surface layer or cross-section of the sample by grinding or cutting to observe the internal structural characteristics of the biofilm [63]. High-resolution CLSM is a widely employed analytical tool for the detection and visualization of the composition of biofilm matrices. Furthermore, it can provide a detailed quantitative characterization of the microstructure within the biofilm, including the biomass and spatial distribution of the microbial population [27,64], and can be used to quantify fluorescence signals [65]. The combination of CLSM and SEM enables the visualization and quantification of the two-dimensional and three-dimensional complex structures of *mycoplasma* biofilms.

#### 2.2.3. Genetic Testing

Biofilms are subject to regulation by several genes, and the application of genome-wide analysis has enabled the development of a more precise assay for the screening of biofilm-associated genes. At present, this method has been employed to identify pivotal genes associated with the regulation of biofilm formation in *M. gallisepticum* that include *manB* (gene encoding phosphomannomutase), *oppA* and *oppD* (components of the oligopeptide permeases complex), *pdh* (pyruvate dehydrogenase), *eno* (enolase), r*elA* ((p)ppGpp synthase/phosphodiesterase), *msbA* (ABC transporter), *deoA* (thymidine phosphorylase), *gapA* (adhesin), and *rpoS* (stationary phase sigma factors) [66]. The genes *oppA* and *oppD* are components of the ATP-dependent oligopeptide transporter complex protein family members that play different roles in bacterial transport over the plasma membrane [66]. DeoA is a key enzyme necessary for the salvage pathway for deoxyribonucleotide synthesis and to use deoxyribonucleosides as a carbon and energy source. In particular, microcolonies of *Mycoplasma hominis* that have entered an energy-deficient state due to aging or other factors will preferentially choose deoxyribonucleotides as an energy source that assists in survival under these environmental stresses, including antimicrobial treatment [67].

Transposon-enabled sequencing is a novel high-throughput sequencing technology that enables the identification of genes essential for bacterial growth in diverse environments and has been utilized in biofilm characterization. This technique has been applied to identify the adhesion gene *VlhA* and the *M. gallisepticum* mutant encoding the adhesion-related factor enolase, as well as the genes involved in EPS synthesis (*manB*, ABC transporter permease, and ABC transporter ATP-binding protein). This technique utilizes an *M. gallisepticum* transposon *Mini-Tn4001SpGm* mutagenesis system [68].

## 3. Correlation Between Biofilm and Resistance of *Mycoplasma*

*Mycoplasma* spp. drug resistance mechanisms include target gene mutations and the expression of efflux pumps. For instance, resistance to fluoroquinolones and aminoglycosides in Tibetan yak *M. bovis* isolates included a single-site base mutation, while doubly mutated isolates led to the emergence of highly resistant strains [69]. In another study, 23/36 strains of *M. anserisalpingitidis* showed a nearly 50% reduction in MIC values after exposure to the efflux pump inhibitor carbon cyanide m-chlorophenylhydrazone (CCCP), thus linking efflux pumps with significant drug resistance [70]. In these contexts, biofilm formation also reduces sensitivity to antibiotics, and drug resistance is significantly enhanced when compared to free-living isolates [29]. At full maturity, antibiotics are typically ineffective against *mycoplasma* biofilms. For example, *M. hyopneumoniae* can persist in biofilms despite exposure to enrofloxacin, tylosin, and florfenicol even at 10× MIC of the cognate planktonic forms [61]. *M*. *synoviae* sensitivity to enrofloxacin, doxycycline, tylosin, and tiamulin decreased following biofilm formation, and the minimal biofilm inhibitory concentration (MBIC) of tylosin increased 4.24-fold compared to the MIC of tylosin [46]. In addition, significantly low negative correlations have been observed between the MBIC of enrofloxacin and biomass and *M. gallisepticum* biofilms that were found to be more resistant to tetracycline and gentamicin than planktonic *M. gallisepticum* [47]. These strains also displayed a greater capacity for biofilm formation, resulting in reduced antibiotic sensitivity [66]. *M. pneumoniae* biofilms formed in vitro at 48 and 72 h exhibited complete resistance to erythromycin concentrations of up to 512 µg mL^−1^ [71]. The MBIC of *U. urealyticum* to tetracycline and levofloxacin after the formation of a biofilm was 4-8-fold or higher than the MIC of its planktonic counterparts, and its quinolone-resistant strains produce more biofilms than the sensitive strains [72,73].

The resistance characteristics of *Mycoplasma* spp. are therefore linked to biofilm formation ability, although exceptions to this have been reported. For example, *M. bovis* biofilms demonstrated no significant resistance to the effects of hygromycin, enrofloxacin, and dafloxacin [31], and no correlation was found between biofilm formation capacity and antibiotic susceptibility in *M. anserisalpingitidis* [32]. In *U. urealyticum*, biofilm formation did not alter its sensitivity to azithromycin and erythromycin [74]. To improve the comparability of anti-biofilm studies, it will be necessary to establish a standardized method to accurately define the biofilm sensitivity endpoint parameters [75]. Therefore, the relationship between *Mycoplasma* spp. biofilms and drug resistance requires further investigation that takes into account the specific *Mycoplasma* species, culture conditions, and biofilm-forming ability.

## 4. Correlation Between Biofilm and Virulence

*Mycoplasmas* are the smallest self-replicating prokaryotes, and their restricted genome size necessitates acquisition of host nutrients. Membrane lipoproteins and proteins are recognized virulence factors in this organism. Invasiveness is mediated by surface adhesins and accessory proteins; capsular polysaccharides and invasion enzymes and biofilm formation all contribute to host survival, environmental adaptation, and immune evasion [76]. Biofilms are therefore a virulence factor in these bacteria and are key components of their pathogenesis [77]. *Mycoplasma* biofilms and planktonic bacteria also differ in immunoreactivity. For instance, six immunoreactive proteins linked to *M. bovis* biofilm formation have been identified, and the HSP-70 family member DnaK displayed the highest level of immunoreactivity and was linked to immune system evasion [74]. In *M. hyopneumoniae*, a significant positive correlation between biofilm formation ability and virulence was identified, and attenuated strains were also low biofilm formers [78]. The P1 adhesin precursor protein in the strongly biofilm-forming *M. gallisepticum* NX-01 has also been identified [66]. The expression of variable membrane surface lipoproteins (Vsps) has also been highly and positively correlated with *M. bovis* biofilm formation. Strains that expressed VspF were poor biofilm formers, while VspO or VspB expressers were strong biofilm formers [31]. This suggested that different strains possess disparate abilities to form biofilms that are most likely associated with variations in the expression of virulence-related genes, including the molecular type. However, the highly pathogenic strain *Mycoplasma mycoides* subsp. *mycoides SC* was unable to produce biofilms in an air/liquid interface model, whereas its cognate strain *Mycoplasma mycoides* subsp. *mycoides LC* was able to form biofilms [31,79]; both the highly virulent strain *M. gallisepticum* S6 and the weakly virulent strain *M. gallisepticum* 6/85 were able to form dense biofilms, indicating that the ability to form biofilms does not necessarily correlate with pathogenicity [47].

Alterations in the survival patterns of *Mycoplasma* spp. during the formation of biofilms may result in variations in the expression levels of virulence genes. *M. pneumoniae* can produce the CARDS toxin that results in host cell vacuolization and activation of the NLRP3 inflammasome [80,81]. However, biofilm growth and CARDS toxin expression in *M. pneumoniae* S1 decrease over time [82]. CARDS toxin and the activities of enzymes associated with the production of H_2_S and H_2_O_2_ were highest during the initial stages of biofilm formation of *M. pneumoniae* and subsequently declined over time [71]. Together, these studies indicated that biofilm formation ability is closely linked to pathogenicity and the ability to establish persistent infections in vivo [83].

## 5. Strategies for Biofilm Control

Biofilms are the leading cause of many persistent and chronic bacterial infections [84]. The emergence of microbial biofilms that adhere to implantable devices or mucosal tissues is a significant global health concern. Biofilms typically increase antibiotic MIC values and may approach levels where the administered drug then reaches toxic levels. A thorough understanding of the mechanism of *mycoplasma* biofilm formation and the relationship between biofilms and drug resistance and virulence demonstrates that timely prevention, control, and removal of biofilms is a key to resolving *mycoplasma* biofilm infections. However, antibiotics that target the *mycoplasma* biofilm have not yet been developed, so finding new strategies to counteract the biofilm is essential for the prevention and treatment of *mycoplasma* biofilm-associated infections.

### 5.1. Physical Methods

When biofilm formation proceeds beyond the adhesion to microcolony and mature biofilm formation, the cells become more recalcitrant to treatment strategies. Therefore, if possible, tackling the developmental process already at the initial adhesion step is most successful to prevent biofilm formation. Disinfection and instrument sterilization and physical methods are commonly used to control biofilms that include ultrasound, ultraviolet light, spray washing, and exposure to elevated temperatures. Chemical reagents such as ethanol and alkaline detergents are also effective against *Mycoplasma* spp. [85]. Biofilm-associated infections associated with adherence to non-biological surfaces can be treated using ultrasound, vortex agitation, and other techniques to isolate adherent bacteria or release bacteria from biofilms [86]. For instance, SMARTF magnetic fluid, which contains iron oxide nano/microparticles, can mechanically remove bacterial cells attached inside flow cells or catheters, and its biofilm removal efficiency is much higher than that of the standard scraping and vortexing methods [87]. Bacterial biofilms exist in drinking water systems [88], and *mycoplasma* can be transmitted to humans and animals via water sources and can pose health risks. Ultrasonic and low-frequency electromagnetic pulse technologies are commonly used to eliminate biofilm in water by inhibiting bacterial adhesion and colonization. Altering the physicochemical properties of adsorption surfaces to inhibit microbial adhesion can also be used and includes enhancing smoothness, wettability, and hydrophilicity or applying surface coatings. The development of new materials with anti-adhesion properties in medical devices can help prevent infections of implanted materials [89].

### 5.2. Biological Formulations

Novel anti-biofilm agents, including plant-derived natural compounds, antimicrobial peptides, and biofilm matrix-degrading enzymes, can play significant roles in preventing bacterial adhesion and suppressing genes associated with biofilm formation. Antimicrobial peptides synthesized from derivatives of the amphipathic, cationic antimicrobial peptide magainin 2 exhibit antibacterial activity, membrane-disrupting properties, and anti-*M. pneumoniae* effects [90]. Sinefungin, a 5′-aminoalkyl analog of S-adenosyl-L-homocysteine, has been demonstrated to inhibit biofilm formation of *M. pneumoniae* and *Streptococcus pneumoniae* in vitro. It functions as a pan-inhibitor against S-adenosylmethionine-dependent methyltransferases and thus holds promise as an inhibitor of *mycoplasma* biofilms [91,92]. Natural compounds such as phenolics, essential oils, terpenoids, plant lectins, alkaloids, peptides, and polyacetylenes have also demonstrated significant anti-biofilm properties [93]. Small molecule inhibitors are compounds that can interact with biomacromolecules such as proteins and nucleic acids, altering their biological activity [94]. The novel narrow-spectrum SM4 and SM9 were screened for their inhibitory effects on the growth of *M. gallisepticum* and were able to demonstrate their antibacterial activity by reducing the invasion of *M. gallisepticum* to host cells and changing the cell membrane integrity of *M. gallisepticum*. Low molecular weight and membrane-active antibacterial properties allow efficacy against *M. gallisepticum* and *M. synoviae* biofilm in vitro [95,96]. SMs showed minimal toxicity on chicken cells and can be used in combination with probiotics, representing a prospective drug to control *M. gallisepticum* biofilms [97]. Additionally, nanoparticles can serve as drug delivery carriers and are capable of penetrating the EPS barrier to target antibiotic delivery, thereby enhancing bioavailability [98]. Zinc oxide nanoparticles (ZnO NPs) show broad antibacterial activity and anti-biofilm activity against pathogenic bacteria and have a sustainable application prospect [99,100]. Aivlosin in combination with ZnO NPs in the treatment is effective in treating *M. gallisepticum* infections in vivo by reducing the level of pro-inflammatory cytokines [101]. Phytochemical-synthesized ZnO NPs, such as Corydalis yanhusuo-ZnO NPs and Albizia chinensis-ZnO NPs, significantly reduce the pathological damage caused by *M. pneumoniae* [102,103]. These demonstrate that the ZnO NPs hold great potential in the field of anti-*mycoplasma* biofilm. In summary, these findings contribute to the development of formulations aimed at eradicating *mycoplasma* biofilms.

### 5.3. Natural Medicines

Organic compounds extracted from natural products, such as phenolics, essential oils, terpenoids, plant lectins, alkaloids, peptides, and polyacetylenes, have also demonstrated significant anti-biofilm properties [93]. For instance, magnolol (5,5′-diallyl-2,2′-dihydroxybiphenyl) is a natural compound with anti-inflammatory, antibacterial, antioxidant, and anticancer properties that significantly inhibits the growth of *M. synoviae*, *M. gallisepticum*, and *M. hyopneumoniae* in vitro and inhibits *M. synoviae* biofilm formation by destroying cell membrane integrity and inhibiting protegenin A production. Energy-related metabolic pathways, including the citrate cycle, glycolysis/gluconeogenesis, and pyruvate metabolism, were significantly disturbed [104,105]. Magnolol has an MIC of 15.63 µg/mL against *M. synoviae*, demonstrating good antibacterial activity, and also has inhibitory effects on other *Mycoplasma* spp. strains. At a dose of 1 mg/kg, magnolol exhibited low cytotoxicity to chicken embryos and significantly reduced the pathogenicity of *M. synoviae*, indicating that it is relatively safe for host cells at the effective dose [105]. Moreover, studies have demonstrated that the volatile components of propolis have significant biological activities, including antioxidant, anticancer, and antiviral properties, with the main compounds characterized as monoterpenes and sesquiterpenes, and exhibit good biocompatibility with normal cells [106,107,108,109,110]. These compounds also have antibacterial potential against Gram-positive and negative bacteria [111,112]. Geopropolis volatile oil (VO) 403 was extracted from *Melipona bicolor schencki*. The primary constituents identified were α-pinene, β-pinene, limonene, and terpinen-4-ol. The MIC of VO 403 was 424 ± 0 μg mL-1 against the *mycoplasma* strains. And it showed 15.25% eradication activity and 13.20% inhibition of *M. pneumoniae* biofilm formation after 24 h at 2× its MIC [113]. A combination of natural medicines and antibiotics has also displayed inhibitory effects on biofilm formation and exhibited synergistic or additive effects [114,115]. As natural active medicines are abundant resources, natural medicines possess characteristics such as high safety, low toxicity, and antibacterial effects. They undoubtedly have promising prospects for long-term development in the prevention and treatment of *Mycoplasma* spp. biofilm-associated infections.

### 5.4. Gene Engineering

Transposons have been widely used in *Mycoplasma* spp. to specifically target and modify genes and have been used successfully to construct biofilm formation defect mutant strains [68,116,117,118]. It is necessary to evaluate their immunogenicity and protective effects, which are expected to be used for the development of live attenuated vaccines. A previous study reported that a transposon mutant library was also used to identify two adhesin genes, *gap*A and *crm*A, required for adhesion and biofilm formation [66,68,119]. The application of genetically modified microorganisms shows great potential in combating persistent bacterial infections and biofilms. A weakly virulent *M. pneumoniae* strain that was constructed and modified through oligonucleotide recombination and Cas9-mediated negative selection was able to express proteins targeting the peptidoglycan of pathogenic bacteria, thereby secreting enzymes that resist biofilms and kill bacteria [117,120,121]. The CRISPR/Cas9-HDR method has also been used to target and knock out genes involved in quorum sensing and adhesion to reduce biofilm formation. This provides a feasible strategy for combating biofilms [122]. Together, these studies provide new perspectives for treating clinically relevant bacterial biofilm infections and demonstrate the potential of novel microbial therapies in the future prevention and treatment of biofilm infections.

## 6. Opportunities and Challenges

Bacterial biofilms are linked to 65% of microbial infections, and 80% of chronic infections are related to bacterial biofilms [123]. These infections are the primary result of biofilms formed on indwelling medical devices and mucosal or soft tissues. For example, the vaginal pathogens *M. genitalium* and *M. homini* can form biofilms. Some species can also combine with other bacterial vaginal pathogens to produce polymicrobial biofilms that allow host persistence [124]. The use of antimicrobial agents to combat biofilms can also result in resistance, and the biofilms cannot be completely eradicated. This is also a primary reason for the high recurrence rate of female reproductive tract infections [125]. The inconsistency between in vivo and in vitro drug sensitivity leads to poor efficacy of antimicrobial therapy, indicating that classical antibiotic susceptibility tests need to consider the impact of biofilms on the tests. It is therefore necessary to develop a standardized method for antibiotic susceptibility testing that specifically targets biofilms. This will improve the consistency between tests and clinical practice [75].

The initial step of biofilm formation does not necessarily require surface adhesion. Bacteria can attach to surfaces in the form of biofilms and can also form biofilm aggregates in a free-floating state [126,127]. This discovery has significant implications for human health and public hygiene. For instance, the biofilm tower structure of *M. pneumoniae* is derived from pre-existing aggregates in vitro, and this contributes to both the spread and persistence of the biofilm lifecycle [128]. Therefore, it is necessary to further verify whether *Mycoplasma* spp. can form non-surface adhesion biofilm aggregates in vivo or in vitro. However, the lack of reliable in vivo imaging technology for biofilm detection is a major obstacle to the study of these biofilms [129]. Therefore, the development of in vivo biofilm detection and tracing methods will promote the study of the interaction between biofilm and tissues and organs. Moreover, *Mycoplasma* spp. lack the classical repertoire of virulence genes common to pathogenic species, and the lack of targeted and efficient genetic tools for genome manipulation hinders the study of the functional characteristics of virulence genes related to pathogenicity [130,131]. This is also one of the important challenges in combating these biofilms. These studies will contribute to an understanding of the relationships between biofilm and clinical pathogenicity.

## 7. Conclusions

*Mycoplasma* biofilms are currently under study in the fields of human and veterinary medicine. *Mycoplasmas* can cause a multitude of diseases in humans and animals. Furthermore, there is a discernible upward trend in the diversity, resistance, and virulence of these organisms. The emergence of multidrug-resistant *mycoplasmas* and their secondary bacterial infectious diseases has constituted a frequent clinical challenge. The ineffectiveness of antimicrobials and vaccines has resulted in significant economic losses for the global farming industry. The formation of biofilms is one of the important reasons for these phenomena, underscoring the urgent need for the development of novel strategies to eradicate *mycoplasma* biofilms. Furthermore, the mechanisms of *mycoplasma* biofilm formation, drug resistance, and virulence factor expression are the current focus of research to better prevent and treat related diseases caused via these biofilms.

## Figures and Tables

**Table 1:** Primary components and functions of biofilms.

Composition	Functions	References
eDNA	Structural component of *Mycoplasma* biofilms, especially those formed on inanimate surfaces (such as glass) where eDNA is concentrated in the basal layer and functions as an attachment surface to enable microcolony formation and can be used as a nutrient source by some species. For example, when *M. bovis* is co-cultured with bovine lung cells, eDNA, as a limiting nutrient, can significantly promote its growth and induce a toxic response to bovine lung cells. If eDNA is insufficient, the growth of *M. bovis* will be limited.	[26,33]
Exopolysaccharides	Multiple functions, including structural support, antibiotic tolerance, and immune evasion. For example, the biofilm of *M. genitalium* contains poly-GlcNAc and promotes antibiotic resistance. The dense polymeric network of poly-GlcNAc can act as a physical barrier, impeding the diffusion of antibiotics into the biofilm. Antibiotics with larger molecular sizes or hydrophilic properties may find it particularly difficult to penetrate this barrier.	[29,34,35]
Protein	Acts as both an adhesion factor and enhances survival and drug resistance. For example, the length of the Vsa protein produced by *M. pulmonis* regulates the ability of mycoplasma to form biofilms by affecting the physical and chemical properties of the cell surface. The truncated part of the Vsa protein mainly occurs in the tandem repeat region at the C-terminus. This structural change directly affects the cell’s sensitivity to complement and its ability to form biofilms. Cells with short Vsa proteins can form biofilms but are sensitive to complement, while cells with long Vsa proteins are resistant to complement but usually cannot form biofilms.	[30,36,37]
Lipid	GC-MS (gas chromatography–mass spectrometry) studies have preliminary evidence for the presence of lipids in *M. genitalium* biofilms, but this was not further explored.	[29]
H_2_O	The largest component in EPS that can maintain moisture in the biofilm and protect cells from desiccation.	[31]

**Table 2:** *Mycoplasma* spp. that are known to form biofilms.

Type	Species	Verified Formation Sites
Human-derived	*Mycoplasma pneumoniae*	abiotic surfaces of either glass or polystyrene [41,42]
	*Ureaplasma urealyticum*	abiotic surface [43]
	*Ureaplasma parvum*	abiotic surface [43]
	*Mycoplasma hominis*	amniotic fluid sludge [44]
	*Mycoplasma genitalium*	polystyrene [29]
	*Mycoplasma salivarium*	an occluded biliary [45]
Animal-derived	*Mycoplasma synoviae*	abiotic surface [46]
	*Mycoplasma gallisepticum*	polystyrene [47]
	*Mycoplasma anserisalpingitidis*	abiotic surface [32]
	*Mycoplasma hyopneumoniae*	abiotic surface [26]
	*Mycoplasma suis*	aortic vessel [48]
	*Mycoplasma bovis*	abiotic surface [31]
	*Mycoplasma putrefaciens*	abiotic surface [31]
	*Mycoplasma yeatsii*	abiotic surface [31]
	*Mycoplasma agalactiae*	abiotic surface [31]
	*Mycoplasma cotewii*	abiotic surface [31]
	*Mycoplasma pulmonis*	mouse tracheal epithelium [49]
	*Mycoplasma meleagridis*	abiotic surface [50]
	*Mycoplasma dispar*	abiotic surface [51]

## Data Availability

The data supporting this systematic review are from previously reported studies and datasets and have been cited.
